# BodyMeta: A comprehensive database for microbes under various pathological and physiological conditions

**DOI:** 10.1016/j.csbj.2025.08.002

**Published:** 2025-08-05

**Authors:** Dong Zhang, Jiamin Hu, Bianli Gu, Bijin Cao, Jinghua Lu, Qiuyue Chen, Lulu Wang, Guangkun Pei, Ze-Xian Liu, Ming-Gang Cheng, Shegan Gao, Xiaoxing Li

**Affiliations:** a Department of Oncology, the First Affiliated Hospital, Sun Yat-sen University, Guangzhou, Guangdong Province 510080, China; b State Key Laboratory of Oncology in South China, Guangdong Provincial Clinical Research Center for Cancer, Sun Yat-sen University Cancer Center, Guangzhou 510060, China; c Henan Key Laboratory of Microbiome and Esophageal Cancer Prevention and Treatment, Henan Key Laboratory of Cancer Epigenetics, Cancer Hospital, The First Affiliated Hospital (College of Clinical Medicine) of Henan University of Science and Technology, Luoyang, China; d Department of Clinical Laboratory, People's Hospital of Bao'an District, Shenzhen Baoan Clinical Medical College of Guangdong Medical University, China; e Institute of Precision Medicine, the First Affiliated Hospital, Sun Yat-sen University, Guangzhou, China

**Keywords:** Microbiota, Human health, Metagenomics, Database, Pathology, Physiology

## Abstract

Microorganisms residing in the gut and other anatomical sites exhibit substantial alterations under both physiological and pathological conditions, which are intricately linked to human health. Consequently, the establishment of a comprehensive metagenomic database encompassing diverse body sites under both pathological and physiological conditions is highly important. In this study, we developed BodyMeta (Body Metagenome Database), an upgraded version of the gutMEGA (gut Metagenome Atlas database), and we expanded the included studies considerably from 182 to 1261. These studies were classified into two categories: 600 literature-based studies without raw data (comprising 107 whole-genome sequencing and 493 16S amplicon sequencing studies) and 661 studies containing 663 raw datasets. We systematically categorized 1842 conditions derived from the 1261 studies into 966 pathological and 879 physiological conditions spanning 31 body sites, with the pathological conditions organized according to MeSH (Medical Subject Headings) terms. We comprehensively annotated the microbial contents, diversities, biomarkers and functional differences of the curated raw 16S amplicon sequencing data. In total, 59010 microbial taxa from literature sources and 40657 from raw datasets were mapped to the NCBI Taxonomy database. Additionally, related information about literature and experiments were compiled within BodyMeta. Collectively, the BodyMeta database provides a comprehensive resource for metagenomic investigations related to both physiological and pathological conditions, which can be freely accessed at https://bodymeta.omicsbio.info/.

## Highlights

● BodyMeta is an update of gutMEGA, expanding the number of included studies from 182 to 1261.
● All included studies were systematically categorized into pathological and physiological conditions, with pathological conditions organized according to MeSH.
● We comprehensively annotated the microbial contents, diversities, biomarkers and functional differences of the curated raw 16S amplicon sequencing data.
● All included microbes included in the database were mapped to the NCBI Taxonomy database.

## 1 Introduction

Microorganisms inhabit not only the intestinal tract but also various other anatomical sites within the human body, including the gastrointestinal tract [1], respiratory system [2], oral cavity [3], urinary tract [4], reproductive tract [5] and skin [6], with each site-specific microbiome playing a distinct yet crucial role in maintaining human health. These microbial communities typically engage in mutualistic symbiosis with the human host during homeostasis, yet this symbiosis can be disrupted under some pathological and physiological conditions, such as colorectal cancer [7], inflammatory bowel disease [8], elevated body mass index (BMI) [9] and consumption of a high-fat diet [10]. Microorganisms that become abnormally enriched under these conditions can contribute to the onset and progression of diseases in multiple ways. For example, Zhang et al.[11] reported that intratumoral *Fusobacterium nucleatum* could recruit tumor-associated neutrophils to promote gastric cancer progression and immune evasion. Yang et al. [10] demonstrated that a high-fat diet induced gut microbiota dysbiosis generates lysophosphatidic acid, which subsequently enhances colorectal cancer (CRC) cell proliferation. Berard et al. [12] observed that microorganisms enriched in bacterial vaginosis could affect epithelial barrier function through the mammalian target of rapamycin (mTOR) pathway. Additionally, there is notable similarity in the composition of the gut microbiota between humans and mice [13], [14]. Due to practical and ethical constraints inherent in human research, numerous studies investigating disease mechanisms and pharmacological effects employ animal models [15], [16], [17], [18], with mice serving as a key model for human gut microbiome research. Collectively, these findings highlight the importance of investigating alterations in the human microbiota under both pathological and physiological conditions to advance human health.

The emergence of high-throughput sequencing technologies, including 16S amplicon sequencing profiles and whole-genome sequencing (WGS) profiles, has significantly advanced our understanding of microorganism—human interactions. The majority of raw sequencing data are deposited in public databases such as the National Center for Biotechnology Information (NCBI) Sequence Read Archive (SRA) [19], the European Nucleotide Archive (ENA) [20], and the DNA Data Bank of Japan (DDBJ) [21]. On the basis of these data, several databases have been established to facilitate the investigation of the relationships between microorganisms and human health. For example, Dai et al. [22] curated 353 human gut metagenome projects and developed GMrepo v2 (data repository for the gut microbiota), a repository dedicated to gut microbiota data that enhances data reusability and enables comparative phenotypic analyses across projects. Qi et al. [23] introduced gutMDisorder v2.0 to facilitate investigations of the dysbiosis of gut microbes in various disorders and therapeutic interventions. The MicrophenoDB [24] was established to detail the relationships among pathogenic microbes, core genes and disease phenotypes through manual curation and data integration. Jin et al.[25] introduced mBodyMap, a database linking microbes within different sites of the whole body to health and diseases. Additionally, in 2020, we established gutMEGA [26] (a database of the human gut metagenome atlas) to host published quantitative gut microbiota data. Despite the valuable insights provided by these resources regarding microorganism–disease associations, they predominantly emphasize pathological conditions while overlooking microorganisms associated with physiological conditions. Consequently, there remains a lack of a comprehensive database that systematically associates microorganisms with both pathological and physiological conditions across diverse body sites.

In this study, we introduce BodyMeta (Body Metagenome), an extensive database that integrates microbial data from 31 distinct body sites under 966 pathological and 879 physiological conditions. We comprehensively annotated the microbial contents, diversities, biomarkers and functional differences of the downloaded raw 16S amplicon sequencing data. From both literature sources and 16S amplicon sequencing datasets, we identified 59,010 and 40,657 microbial taxa, respectively, all of which were mapped to the NCBI Taxonomy database. Additionally, we collected related information about the literature and experiments, developed interactive charts and implemented diverse search and browsing functionalities to facilitate user access to microbial information associated with both pathological and physiological conditions.

## 2 Methods

### 2.1 Collection of raw data

We searched the PubMed database with the following keywords: ‘gut’, ‘oral’, ‘respiratory tract’, ‘microbiota’, ‘microbiome’, etc., from 2006 to 2024. After including newly acquired studies and gutMEGA investigations, we obtained 1261 studies, representing a considerable increase (Table 1). Given the increasing importance of shotgun metagenomic sequencing or achieving higher taxonomic and functional resolution, we have incorporated WGS data extracted from the literature into our database to enhance its comprehensiveness while maintaining consistency across studies. For newly acquired 16S amplicon sequencing studies, we further categorized the data into two types: (1) those providing defined phenotypes and publicly accessible raw sequencing data, and (2) those lacking either defined phenotypes or accessible sequencing data. For the first group, we utilized Aspera (a high-speed data transfer tool) to download raw reads from the EBI ENA database [20] and then verified data integrity, resulting in the collection of 663 datasets. The corresponding metadata were downloaded and manually processed via R scripts. For studies lacking either defined phenotype information or publicly available raw 16S amplicon sequencing data, we extracted microbial data directly from the literature. Furthermore, we collected related information about literature and experiments, such as sample location, sample type, and sequencing platform.

**Table 1:** The key features of BodyMeta and comparison with gutMEGA on data contents.

Database	Study type	Sample location	Host species	Data type	Study numbers
gutMEGA	16S	Gut	Human	Literature-based	152
gutMEGA	WGS	Gut	Human	Literature-based	30
BodyMeta	16S	Various body sites	Human; Mouse	Literature-based	493
BodyMeta	WGS	Various body sites	Human; Mouse	Literature-based	107
BodyMeta	16S	Various body sites	Human; Mouse	Raw data-based	661

### 2.2 Classification of conditions

We ultimately compiled a total of 1842 conditions across 31 body sites. To enhance user navigation, we further categorized the conditions into pathological conditions and physiological conditions. The pathological conditions comprised a spectrum of diseases affecting different systems and were organized according to the MeSH terms. For the physiological conditions, instead of using a standardized ontology, we manually classified the studies based on their primary research focus to facilitate practical user retrieval and dataset utilization. These classifications included non-disease-related factors such as body weight, age, ethnicity, diet, exercise, environmental exposures, and other physiological or lifestyle-related factors commonly investigated in microbiome research. Additionally, studies investigating the impacts of specific drugs, herbs, probiotics, and prebiotics on health or disease prevention were also included under physiological conditions. In summary, a total of 1842 conditions were classified into 23 types of pathological and 19 types of physiological conditions.

For the physiological conditions, instead of using a standardized ontology, we manually classified the studies based on their primary research focus to facilitate practical user retrieval and dataset utilization. The physiological conditions included nondisease-related factors such as weight, age, ethnicity, and environmental factors. Additionally, studies investigating the therapeutic impacts of specific herbs, probiotics, and prebiotics on diseases have classified these effects as physiological conditions.

### 2.3 Data processing

We systematically processed the 663 raw 16S amplicon sequencing datasets with advanced analytical tools. The raw 16S amplicon sequencing data and associated metadata were imported into the QIIME2 pipeline [27] (version 2023.7) to obtain amplicon sequence variants (ASVs) instead of operational taxonomic units (OTUs). After importing raw sequence data, DADA2 [28] integrated into QIIME2 was used for quality control including quality filtering, paired-end sequence merging, denoising and chimera removal. Feature classifiers [29] were trained against the SILVA database (version 138.1) [30] for various hypervariable regions of 16S rRNA genes and subsequently applied to ASVs to obtain taxonomic abundance. However, due to the limited resolution of 16S amplicon sequencing, species-level classification was not applied to 16S datasets, regardless of whether the data were obtained from raw sequencing data or extracted from the literature. After that, downstream analyses were conducted in accordance with methodologies delineated in a recent publication [31]. First, we performed alpha-diversity and beta-diversity analyses. We then employed linear discriminant analysis effect size (LEfSe) analysis [32] to identify associated microbes (linear discriminant analysis score> 2). Next, we performed functional prediction analysis via PICRUSt2 [33] software to predict the MetaCyc [34] pathway abundances of the samples under each condition. The ALDEx (ANOVA-Like Differential Expression) method [35], implemented in ggpicrust2 software [36], was subsequently used to assess differential pathway abundance.

### 2.4 Taxon rematch

After obtaining the microbes from literature and raw sequencing data, we used the Taxonkit [37] software to standardize all taxonomic annotations by matching the microbes to the NCBI Taxonomy database [38], retrieving the taxonomic ranks and the corresponding NCBI TaxonIDs for each microbe. To complement NCBI Taxonomy and account for newly classified or uncultured genomes, we incorporated annotations from the Genome Taxonomy Database (GTDB, release r226) [39]. For each microbial taxon in our database (based on NCBI classification), we identified all genomes in the GTDB metadata annotated with the same taxon name at the corresponding rank. We then extracted the GTDB-assigned taxonomy for these genomes and summarized the classification results within that rank. This approach allowed us to quantify the taxonomic diversity recognized by GTDB under a single NCBI taxon and to highlight potential reclassifications or refinements.

### 2.5 Database construction

All the data in BodyMeta were stored in a MySQL database, and the website was deployed via an Apache server. For the front end, bootstrap, an open-source user interface framework based on HTML, CSS, and JavaScript, was used to build the basic layout of the website. Interactive functions were implemented via JavaScript and its tool library, JQuery. Data processing at the back end was performed via the GO programming language, and the interaction between the front and back ends was achieved via asynchronous JavaScript and XML (AJAX). In addition, to ensure stable and adaptive service, we tested the BodyMeta website via various web browsers, including Mozilla Firefox, Google Chrome, Microsoft Edge, and the 360 Browser.

## 3 Results

### 3.1 Workflow and composition of BodyMeta

**Fig. 1:**
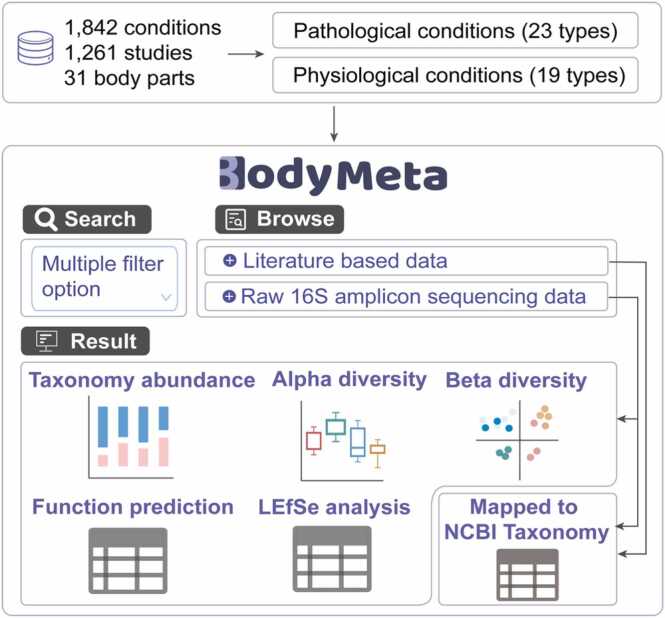
Overall workflow and composition of BodyMeta.

Overall, 1842 conditions were classified into 19 types of physiological conditions and 23 types of pathological conditions (Fig. 1). For the raw 16S amplicon sequencing datasets, we comprehensively annotated the microbial contents, diversities, biomarkers and functional differences and provided interactive charts. Additionally, the microbes identified through LEfSe analysis, alongside those derived from the literature, were systematically mapped to the NCBI Taxonomy database.

**Fig. 2:**
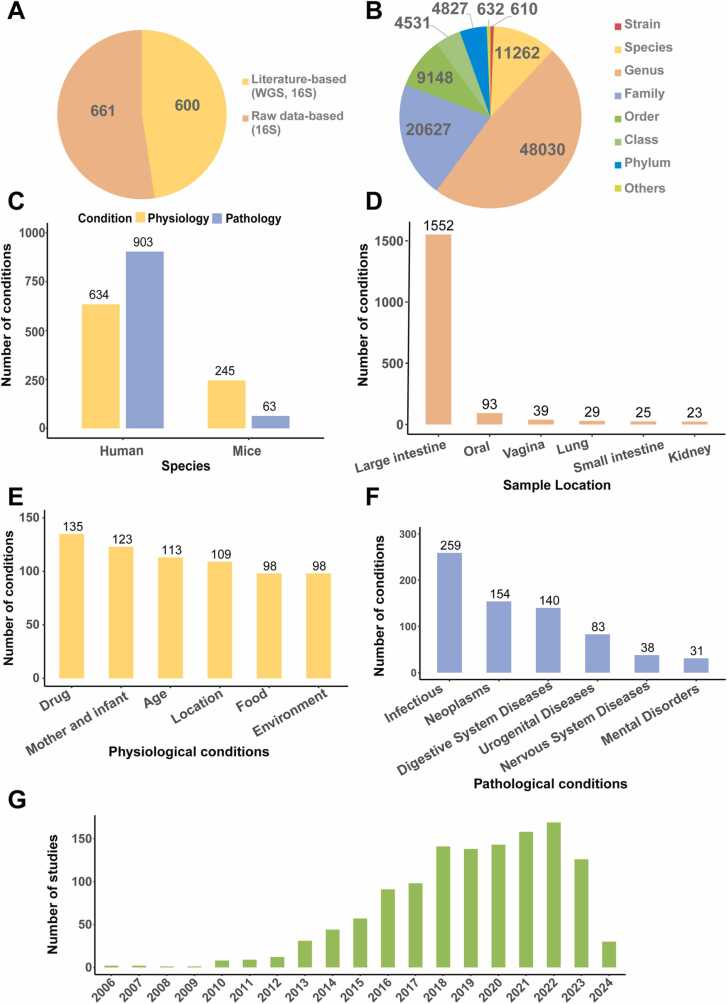
Statistics of the data composition in BodyMeta. (A) The classification of all included studies. (B) The distribution of microbes at different classification levels. (C) Bar plot showing the number of physiological and pathological conditions studied in human and mouse microbiome datasets. (D) The top six sample locations of all included conditions. (E) The top six physiological conditions of all included conditions. (F) The top six pathological conditions of all included conditions. (G) Year distribution of all included studies.

On the whole, the database comprised 1261 studies, stratified into 600 literature-based studies and 661 raw data-based studies (Fig. 2A). The distribution of all microbes obtained from both sources is illustrated in a pie chart (Fig. 2B), with the majority classified at the genus level. Statistical evaluation of the conditions represented in BodyMeta revealed a predominance of human-associated conditions compared to those related to murine models (Fig. 2C). Moreover, the analysis indicated a research bias toward large intestinal samples (Fig. 2D), highlighting the significance of the gut microbiota in human health. The six most frequently studied physiological conditions include drug exposure, maternal and infant factors, age, geographic location, diet, and environmental influences (Fig. 2E). In contrast, the leading pathological conditions comprise infection, neoplasms, digestive system diseases, urogenital diseases, nervous system diseases and mental disorders (Fig. 2F). A longitudinal analysis of publication trends demonstrated a steady rise in the number of metagenomic studies over time, highlighting the increasing scientific focus on the interplay between the microbiota and human health (Fig. 2G).

### 3.2 Query function and result presentation of BodyMeta

**Fig. 3:**
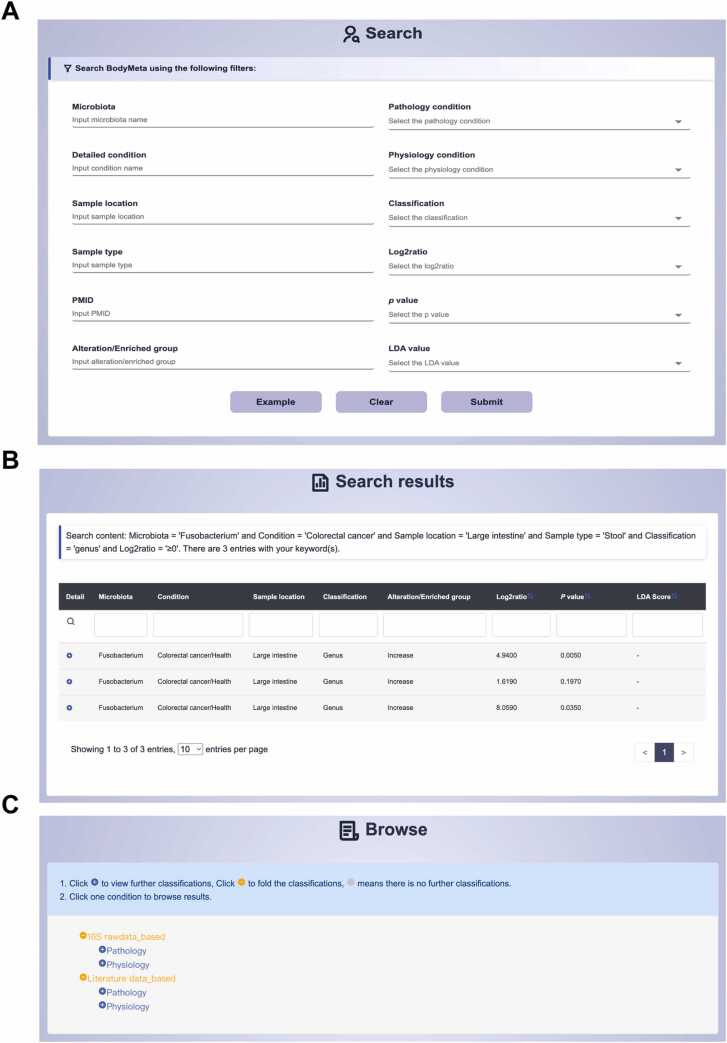
Main pages on BodyMeta to query results. (A) Advanced search function on the ‘Search’ page. (B) Search results for a given example. (C) Browse function for all the included conditions on the ‘Browse’ page.

The BodyMeta database provides researchers with different querying modes to facilitate user-friendly access to microbial information. Users can utilize the basic quick search tool available on the ‘Home’ page, the advanced search functionality on the ‘Search’ page, or the browsing interface on the ‘Browse’ page (Fig. 3A-C) to query the database. The advanced search provides multiple filtering options to refine results. Users can select a specific pathological or physiological condition, or directly input the detailed condition name of interest for customized queries. Additionally, users can further refine their search by specifying the sample location and sample type associated with each study. Further filtering based on specific parameters such as PMID, Log2ratio and p value is also supported.

On the ‘Browse’ page, all conditions have been systematically organized into two primary parts: ‘16S-based’ and ‘Literature-based’ (Fig. 3C). The ‘16S-based’ part encompasses microbial data obtained from raw 16S amplicon sequencing data, while the ‘Literature-based’ part compiles information from the newly acquired literature and the gutMEGA database. Within each part, conditions are further divided into physiological and pathological categories, thereby facilitating users’ ability to efficiently identify and access pertinent research topics. Moreover, identical conditions are grouped into the same option, each accompanied by a description of the sample type and location.

**Fig. 4:**
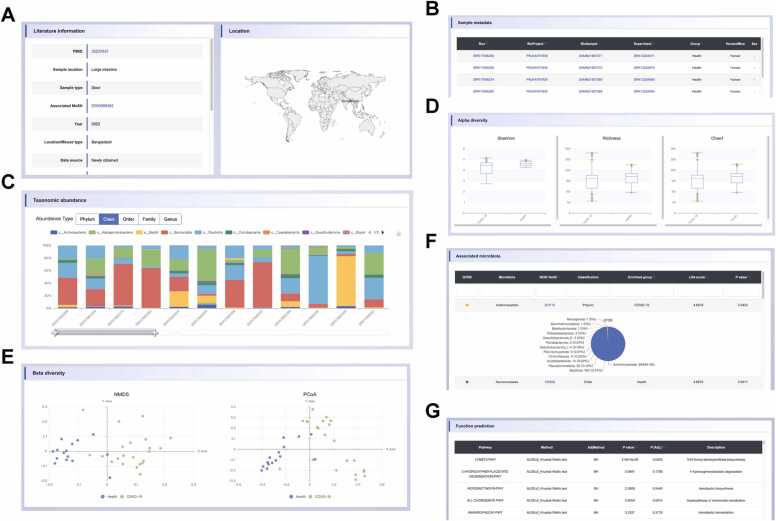
Results of the ‘16S-based’ part in BodyMeta. (A) The detailed literature information, geographic location of participants or mouse type of relevant study. (B) Information for each sample under such conditions, including NCBI accession numbers and other detailed information. (C) Stacked bar plot displaying the taxonomic abundance of each sample at different classification levels; each color represents one microbe. (D) Boxplot displaying alpha diversity via three indices. (E) Scatterplots displaying beta diversity, with each dot representing one sample. (F) Table displays results of associated microbes; pie chart shows GTDB annotation results for each microbial taxon. (G) Table showing the results of ALDEx differential analysis of MetaCyc pathways predicted by PICRUSt2.

Upon selection of a specific condition within the ‘16S-based’ section, users are presented with eight distinct sections. The ‘Literature information’ offers relevant literature details, including the PMID, sample location, sample type, sequencing technology, sequencing platform, etc. (Fig. 4A). The ‘Location’ or ‘Mouse type’ section provides information on the geographic locations of human study participants or the strains of mice utilized in the studies (Fig. 4A). The ‘Sample metadata’ section includes grouping information and unique identifiers (‘Run’, ‘BioProject’, ‘Biosample’, and ‘Experiment’), all of which are hyperlinked to the NCBI database (Fig. 4B). The ‘Taxonomic abundance’ section illustrates the microbial composition of each sample at five classification levels: phylum, class, order, family and genus (Fig. 4C). The ‘Alpha diversity’ section provides the microbial diversity measured by the Shannon, Richness and Chao1 index visualized through boxplots (Fig. 4D). The ‘Beta diversity’ section provides principal coordinate analysis (PCoA) and non-metric multidimensional scaling (NMDS) analysis results on the basis of Bray-Curtis dissimilarities, depicted via scatterplots (Fig. 4E). The ‘Associated microbiota’ section identifies associated microbes of each group, with all taxa mapped and linked to the NCBI Taxonomy database. For each associated microbe, the “GTDB” feature delineates the taxonomic correspondence between the NCBI and GTDB classification systems at the specified rank, with distributions visualized through pie chart (Fig. 4F). Lastly, the ‘Function prediction’ section provides differential MetaCyc pathways predicted by PICRUSt2 and analyzed using the ALDEx method (Fig. 4G). In contrast, when a condition is selected within the ‘Literature-based’ part, only three sections are displayed: ‘Literature Information’, ‘Location’ or ‘Mouse type’, and ‘Associated microbiota’, which correspond to the analogous categories available in the ‘16S-based’ part.

### 3.3 Example of BodyMeta usage

**Fig. 5:**
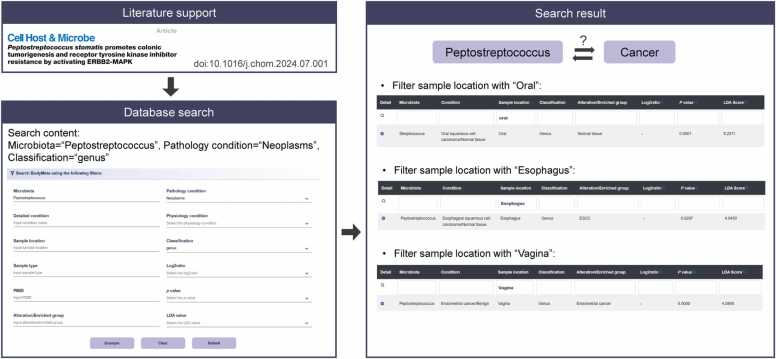
An example of BodyMeta usage.

To better understand the usage of BodyMeta, we provide an example of usage (Fig. 5). Recent research has demonstrated that *Peptostreptococcus stomatis* was enriched in colorectal cancer and accelerates colonic tumorigenesis by inducing cell proliferation, suppressing apoptosis, and impairing gut barrier function [40]. To explore potential pan-cancer associations involving *Peptostreptococcus*, we queried the BodyMeta database using ‘Peptostreptococcus’ as the microbiota keyword, with genus-level classification and ‘Neoplasms’ selected as the pathological condition. After applying relevant filters, we observed that *Peptostreptococcus* was significantly enriched in the buccal mucosa of patients with oral squamous cell carcinoma, in esophageal tissues of patients with esophageal squamous cell carcinoma, and in vaginal secretions of patients with endometrial carcinoma. These preliminary observations indicate a possible link between *Peptostreptococcus* and tumorigenesis across multiple human anatomical sites. While these findings do not establish a causal relationship between the microbiota and cancer development, they provide supportive evidence for further investigations in this field. This demonstrates the capability of the BodyMeta database to identify potential microbial associations across multiple body sites, offering insights into their coordinated roles under different physiological or pathological conditions

## 4 Discussion

Recent research has moved beyond examining solely the relationships between gut microbiota and diseases, recognizing that microbial communities across various anatomical sites—including the oral cavity, lungs, skin, and vaginal tract—are intricately linked to human pathological phenotypes and physiological functions. The emergence of high-throughput sequencing technologies has facilitated the accumulation of extensive metagenomic datasets. Despite the development of numerous metagenomic databases, there remains a need to explore the dynamic changes in microbiota across different body sites under both pathological and physiological states. In response to this gap, we have developed BodyMeta, an upgraded version of the gutMEGA database, significantly expanding the number of incorporated studies from 182 to 1261. BodyMeta now serves as a comprehensive database that encompasses a broad spectrum of metagenomic investigations under 966 pathological and 879 physiological conditions.

The BodyMeta database consolidates a broad spectrum of microbiological research, encompassing common conditions such as diabetes mellitus and inflammatory bowel disease, alongside rarer genetic disorders including Rett syndrome and Fragile X syndrome. It covers diverse physiological states, ranging from high-fat and ketogenic dietary regimens to investigations of microbial communities in cadaveric samples. Each condition has been systematically categorized to facilitate efficient and precise retrieval of associated research by users. For studies based on raw data, comprehensive analytical results are provided, detailing the microbial composition, diversities, biomarkers and functional differences, thereby enabling users to attain a comprehensive understanding of the microbial landscape. Furthermore, BodyMeta offers a robust suite of access functionalities designed to ensure efficient and accurate information retrieval. Collectively, the BodyMeta database was designed to serve as a reliable and comprehensive resource for researchers seeking to explore microbial variations across different anatomical sites under a wide spectrum of physiological and pathological conditions.

To enhance its role as a comprehensive repository encompassing diverse physiological and pathological conditions, the BodyMeta database has several areas for future development. Notably, the analysis of raw shotgun metagenomic data has the potential to offer more intricate details about the microbial composition, including the detection of fungi and viruses, as well as providing a more exhaustive functional characterization. By effectively removing various confounding factors and integrating disparate datasets for the same conditions, more robust and generalizable conclusions can be achieved. Moving forward, we plan to update the BodyMeta database on an annual basis to maintain its currency and to continuously incorporate the latest advancements in the field.

## Authors’ contributions

XX.L., SG.G. and MG.C. designed and supervised the experiments. D.Z. performed the experiments and data analysis; JM.H., BL.G. and BJ.C. developed the database. JH.L., QY.C. and LL.W. contributed to the data collection and database development. D.Z. wrote the manuscript with contributions from all the authors. All the authors reviewed the manuscript.

## CRediT authorship contribution statement

**Guangkun Pei:** Formal analysis. **Lulu Wang:** Formal analysis. **Ming-Gang Cheng:** Investigation. **Ze-Xian Liu:** Conceptualization. **Xiaoxing Li:** Investigation, Funding acquisition, Conceptualization. **Dong Zhang:** Formal analysis, Data curation. **Shegan Gao:** Investigation. **Bianli Gu:** Formal analysis, Data curation. **Jiamin Hu:** Investigation, Data curation. **Bijin Cao:** Investigation. **Qiuyue Chen:** Data curation. **Jinghua Lu:** Data curation.

## Declaration of Competing Interest

The authors declare that they have no competing interests.

## Data Availability

The data are available on the “Download” page of BodyMeta at https://bodymeta.omicsbio.info. All the data supporting the findings of this study can be obtained from the corresponding author upon reasonable request. All R scripts used for metadata processing and analysis are openly accessible on GitHub (https://github.com/lzxlab/BodyMeta).
